# Analytical validation of a next generation sequencing liquid biopsy assay for high sensitivity broad molecular profiling

**DOI:** 10.1371/journal.pone.0193802

**Published:** 2018-03-15

**Authors:** Vincent Plagnol, Samuel Woodhouse, Karen Howarth, Stefanie Lensing, Matt Smith, Michael Epstein, Mikidache Madi, Sarah Smalley, Catherine Leroy, Jonathan Hinton, Frank de Kievit, Esther Musgrave-Brown, Colin Herd, Katherine Baker-Neblett, Will Brennan, Peter Dimitrov, Nathan Campbell, Clive Morris, Nitzan Rosenfeld, James Clark, Davina Gale, Jamie Platt, John Calaway, Greg Jones, Tim Forshew

**Affiliations:** 1 Research and Development, Inivata Ltd, Granta Park, Cambridge, United Kingdom; 2 Product Development, Inivata Inc, Research Triangle Park, North Carolina, United States of America; 3 Clinical Development, Inivata Inc, Research Triangle Park, North Carolina, United States of America; 4 Clinical Laboratory Operations, Inivata Inc, Research Triangle Park, North Carolina, United States of America; CNR, ITALY

## Abstract

Circulating tumor DNA (ctDNA) analysis is being incorporated into cancer care; notably in profiling patients to guide treatment decisions. Responses to targeted therapies have been observed in patients with actionable mutations detected in plasma DNA at variant allele fractions (VAFs) below 0.5%. Highly sensitive methods are therefore required for optimal clinical use. To enable objective assessment of assay performance, detailed analytical validation is required. We developed the InVisionFirst^™^ assay, an assay based on enhanced tagged amplicon sequencing (eTAm-Seq^™^) technology to profile 36 genes commonly mutated in non-small cell lung cancer (NSCLC) and other cancer types for actionable genomic alterations in cell-free DNA. The assay has been developed to detect point mutations, indels, amplifications and gene fusions that commonly occur in NSCLC. For analytical validation, two 10mL blood tubes were collected from NSCLC patients and healthy volunteer donors. In addition, contrived samples were used to represent a wide spectrum of genetic aberrations and VAFs. Samples were analyzed by multiple operators, at different times and using different reagent Lots. Results were compared with digital PCR (dPCR). The InVisionFirst assay demonstrated an excellent limit of detection, with 99.48% sensitivity for SNVs present at VAF range 0.25%-0.33%, 92.46% sensitivity for indels at 0.25% VAF and a high rate of detection at lower frequencies while retaining high specificity (99.9997% per base). The assay also detected *ALK* and *ROS1* gene fusions, and DNA amplifications in *ERBB2*, *FGFR1*, *MET* and *EGFR* with high sensitivity and specificity. Comparison between the InVisionFirst assay and dPCR in a series of cancer patients showed high concordance. This analytical validation demonstrated that the InVisionFirst assay is highly sensitive, specific and robust, and meets analytical requirements for clinical applications.

## Introduction

It has been shown more than 20 years ago that some cancer mutations can be detected non-invasively through analysis of samples including blood plasma, urine, stool and sputum [1–6]. Circulating tumor DNA (ctDNA) is believed to enter a patient’s blood plasma largely through turnover of cancer cells and subsequent release of the resultant fragmented DNA into circulation. Early attempts to analyze this ctDNA were restricted to methods that focused on a small number of genomic changes with relatively limited sensitivity. It is now known that a significant fraction of mutations, especially in earlier stage cancer are present at extremely low variant allele fractions (VAF) in the blood.

The development of methods including digital PCR (dPCR) and its derivatives such as droplet-based digital PCR (ddPCR) subsequently enabled the sensitive and quantitative analysis of ‘hotspot’ mutations or individual mutant alleles [7–9]. These more sensitive methods demonstrated the potential of using ctDNA for a range of applications including cancer prognostication, treatment selection, monitoring and even early detection [9–11]. They were still however limited to assessing just a small number of changes.

We demonstrated for the first time in 2012 the ability to use next generation sequencing (NGS) of gene panels to detect solid tumor mutations through sequencing a patient’s cell free DNA (cfDNA) and to monitor the VAF of multiple mutations in serially collected plasma samples over time [12]. The initial version of our assay using TAm-Seq^®^ technology covered 6 genes and had 97% sensitivity and specificity for detecting single nucleotide variants (SNVs) and indels at 2% VAF and above and reported mutations down to 0.14% VAF. This demonstration was rapidly followed by examples of a range of different NGS approaches including hybrid capture and molecular barcoding that could be applied to broadly analyze ctDNA with varying performance characteristics [13–15].

The area where ctDNA analysis is most rapidly entering clinical use is in the molecular stratification of patients for treatment where tissue is limited, unavailable or of insufficient quality; most notably for non-small cell lung cancer (NSCLC) patients. This is due to the complexity of a lung biopsy, the risk and associated costs and the availability of appropriate effective targeted agents for treatment of NSCLC patients. The first assays to gain regulatory approval for testing in this setting were the therascreen^®^ EGFR RGQ PCR kit and cobas^®^ *EGFR* Mutation Test v2 assays which use real-time PCR for the qualitative detection of *EGFR* exon 19 deletions, L858R, T790M and other mutations in *EGFR*. Positive ctDNA results can be used to determine which NSCLC patients are eligible for treatment with 1^st^- or 3^rd^-generation *EGFR* inhibitors [16]. Due to the technology used however, these assays are less sensitive than dPCR and can only assess a limited number of mutations, reducing the number of patients these assays will successfully stratify to treatment.

**Fig 1:**
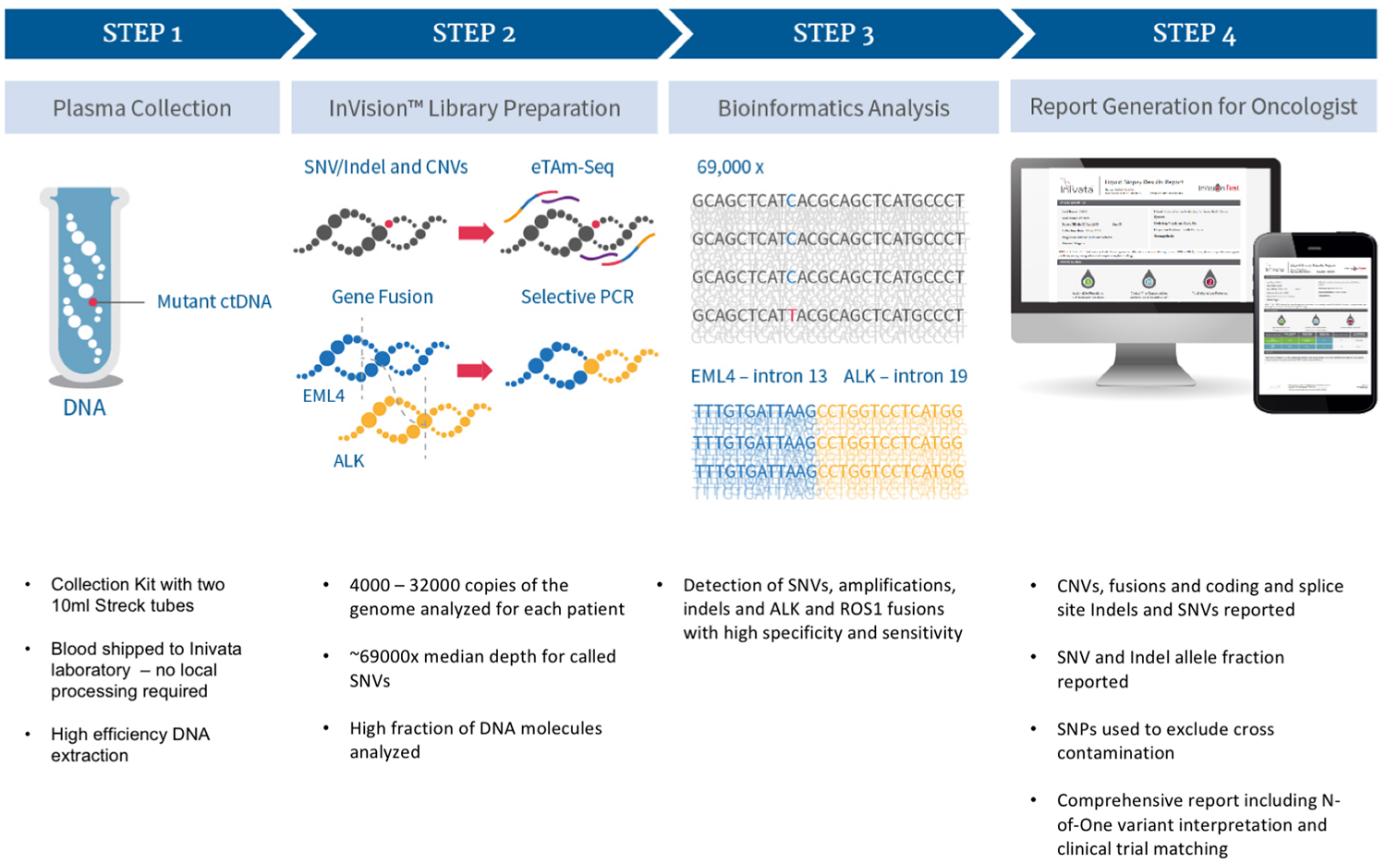
Overview of the InVisionFirst workflow.

To enable broad and highly sensitive ctDNA analysis we have developed eTAm-Seq technology, a significantly enhanced version of our original TAm-Seq technology (Fig 1). We previously described the development of an earlier version of this enhanced assay, which covered 35 genes and could detect SNVs, indels and copy number variations (CNVs) [17]. Here we describe the analytical validation of the InVisionFirst assay which utilizes this technology and has been updated to cover 36 genes for a range of SNVs, indels, CNVs and gene fusion events including the key mutations in *EGFR* and *ALK* and *ROS1* fusions (S1 Table). InVisionFirst is an NGS assay designed to detect the key actionable somatic NSCLC mutations in ctDNA, released into the blood stream of NSCLC patients which, when combined with standard clinical observations, can be used by the clinician to guide a patient to therapy. Based on the previously published NSCLC mutation spectrum, 94% of patients contain at least one mutation within the 36 genes targeted [18].

There are a growing number of ctDNA assays being developed based on different technologies, with different performance characteristics and differing levels of concordance with tissue [15,19]. It is therefore critical that analytical validation studies are executed and performance testing schemes developed to determine the functional characteristics of each assay to enable clinicians to select the most suitable assay for their patient. In the current study, we first describe the assessment of the InVisionFirst assay’s ability to call SNVs, indels, amplifications and gene fusions using contrived material and blood from donors not known to have cancer. We then compare our ability to call changes in NSCLC patients’ blood with that of digital PCR. Finally, we demonstrate that the InVisionFirst assay gives concordant results whether blood is drawn in Streck Cell-Free DNA Blood collection tubes (Streck BCT) or EDTA tubes and we show, concordant with previously published results, that when DNA containing mutations was spiked into blood drawn into Streck BCT then the mutant allele fraction stayed stable for at least 10 days.

## Results

### SNV detection sensitivity, repeatability and reproducibility

To determine the ability of the InVisionFirst assay to call mutations at different allele fractions and thus its limit of detection (LoD) which we have defined as the point where we would detect a mutation ≥90% of the time (LoD90), a dilution series was created of sheared Tru-Q7 reference DNA in Tru-Q0 (both obtained from Horizon Discovery). Details of this and subsequent contrived materials are described in detail in the Materials and Methods section. Tru-Q7 contains 39 validated mutations that are covered in our targeted sequencing region, and 32 of these are SNVs present at low VAF, predominantly between 1%-1.3% (S2 Table). The dilution series created 5 different samples, containing respectively the majority of mutations at the following VAF levels: 1%-1.3%, 0.5%-0.65%, 0.25%-0.33%, 0.13%-0.16% and 0.06%-0.08%. Samples from this dilution series was analyzed multiple times by three operators, each using two different Lots of reagents. Full details of this and subsequent designs are available in S3 Table.

**Fig 2:**
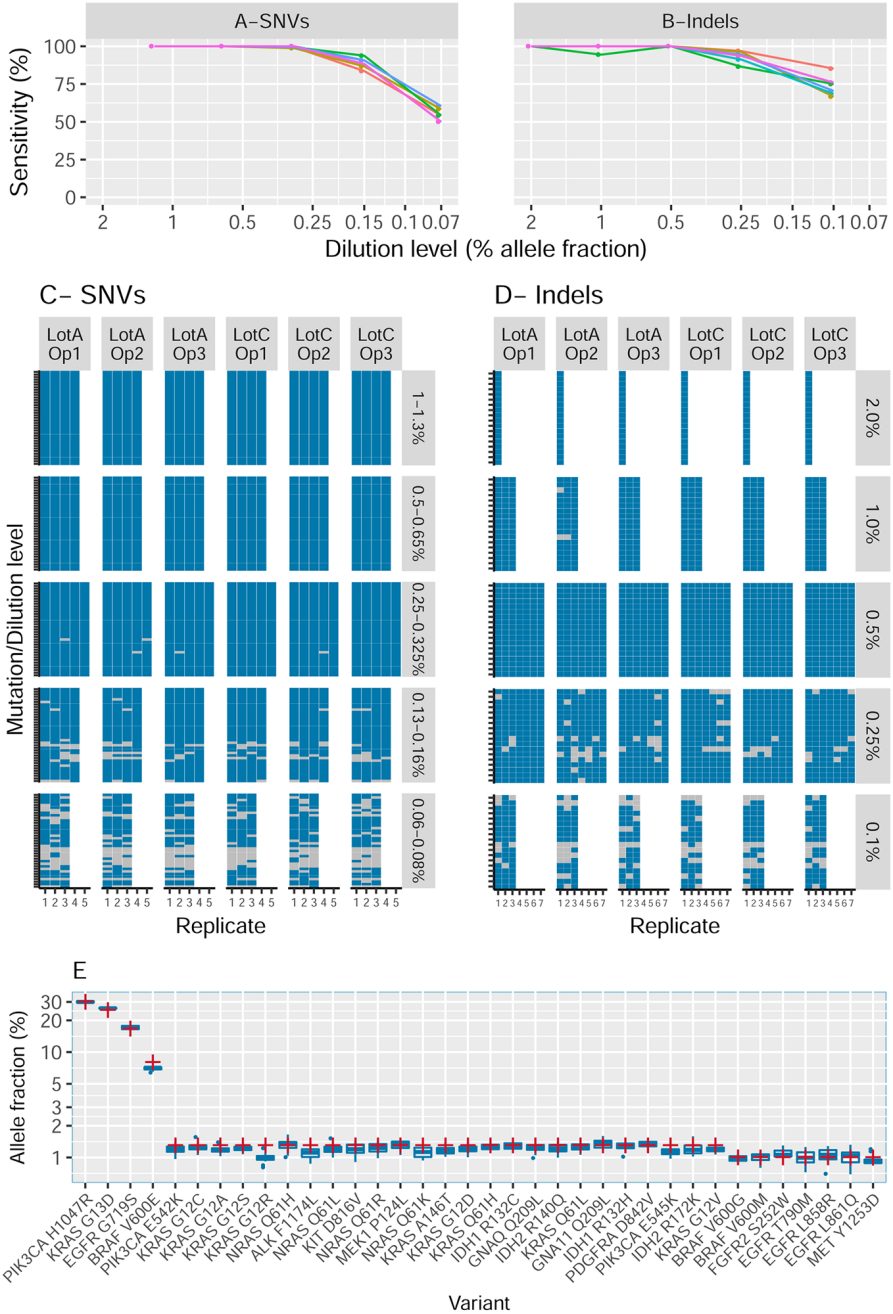
Sensitivity for SNVs (A, C) and indels (B, D). **A** and **B** show the sensitivity as a function of the allele fraction of the reference mutations. Each line represents a different operator/Lot combination. **C** and **D** show the full set of calls for all combinations of dilution/variant (vertical) and repeat/operator/lot (horizontal). Blue rectangles represent mutations that were detected and grey represents those missed. Panel E shows for SNVs the estimated allele fraction compared between InVisionFirst (blue box-plots) and the reference as estimated by Horizon using ddPCR (red crosses).

100% of SNVs with an expected VAF of 0.5% and above were detected across all runs, all operators and all reagent Lots. For SNVs at VAF in the range 0.25%-0.33%, 99.48% were detected. 88.93% of SNVs were detected at the VAF range of 0.13%-0.16%, and 56.25% were detected at the VAF range 0.06%-0.08% (Fig 2A, S4 Table). This confirmed the LoD of our assay to be 0.25% VAF. A complete table describing all expected calls and whether they were made, along with depth of coverage is available (S5 Table). In total, there were 3,498 mutation calls made at low VAF (≤1.3%) in this SNV section of the study. The median depth of sequencing for all the detected mutations was 69,061x and the lowest depth at which any of the 3,498 mutation calls were made was 10,223x. Coverage was extremely even, with 95% of calls having a depth no less than half the median and 99.9% of calls having a depth no less than 0.2x of the median.

Mutation calls between replicates, operators and reagent Lots showed high repeatability and reproducibility with all 32 mutations detected in all replicates at 0.5% VAF and above and no noticeable difference within or between operators or Lots at 0.25%-0.33% VAF (Fig 2C). To extend our analysis across a broader set of mutations we assessed a total of 43 unique samples containing a total of 605 unique variants at or above the LoD (S6 Table). We detected all SNVs giving a Positive percentage agreement (PPA) of 100.0% at ≥0.25% VAF.

Combining the replicates of the InVisionFirst assay, the average estimated VAF for the 36 validated SNVs closely correlated with the expected frequencies as stated by Horizon for the undiluted Tru-Q7 DNA (Pearson squared correlation coefficient R^2^ = 0.9987) (Fig 2E).

### Indel detection sensitivity, repeatability and reproducibility

To assess the InVisionFirst assay’s ability to call indels, a custom reference material was created by SeraCare containing eighteen indels targeted by our panel ranging from -24bp to +12bp (S7 Table). Five separate samples were produced by SeraCare with all eighteen indels present at one of five different levels; 2%, 1%, 0.5%, 0.25% or 0.1% VAF. All five of these samples were analyzed multiple times by three operators each using two different Lots of reagents (S3 and S7 Tables).

For the 2%, 1% and 0.5% VAF all but 3 of the 1188 expected indels were detected (99.7%). At a VAF of 0.25%, 92.46% of indels were detected, whilst at 0.1% VAF, 234 out of an expected 324 indels (72.22%) were detected (Fig 2B and S8 Table). A complete table of all expected indels and whether they were detected are available in S9 Table.

As with the SNV calling, the sensitivity of the assay did not vary within runs, between operators or between reagent Lots demonstrating high assay sensitivity, repeatability and reproducibility (Fig 2D).

To extend indel analysis across a broader set of unique samples and mutations we assessed a total of 31 unique samples containing a total of 115 variants at or above our LoD (≥0.25% VAF) demonstrating a PPA of 97.4% (S6 Table).

### Fusion gene detection sensitivity, repeatability and reproducibility

The InVisionFirst assay detects the DNA breakpoints that create the common *EML4*-*ALK* and *ROS1* gene fusions. Due to the scarcity of DNA samples with such breaks, three separate approaches were used to assess our sensitivity to detect these fusions. Fragmented cell line DNA was created (Horizon Discovery) with one *EML4*-*ALK* and one *SLC34A2-ROS1* fusion. Dilutions with these fusions at five different levels were created (VAF of 1%, 0.5%, 0.25%, 0.13% and 0.06%) and a similar replication strategy as used for SNVs and indels was undertaken (S3 Table).

**Fig 3:**
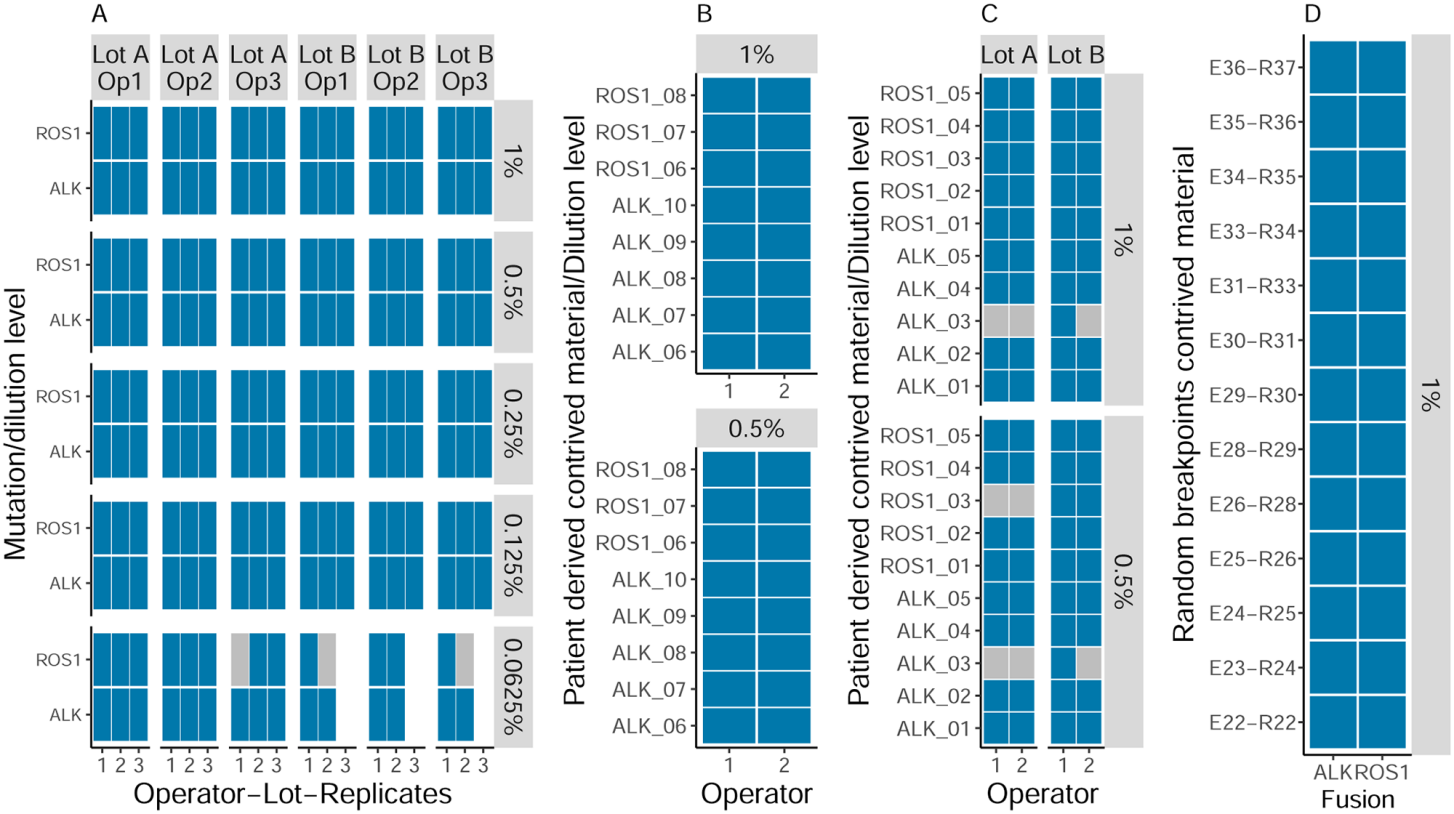
Fusion sensitivity analysis. Blue rectangles represent fusions that were detected and grey represents those missed. (A) Dilution of Horizon reference material containing 2 fusions (ALK and ROS1) across dilution levels (vertical) and operator/lot (horizontal), (B) Set 1 of contrived material based on published DNA breakpoints (AF 1% and 0.5%, 2 operators), (C) Set 2 of contrived material based on published DNA breakpoints (AF 1% and 0.5%, 2 operators, 2 reagent lots) and (D) Contrived material based on randomly generated fusion breakpoints. Different operators performed different parts of this fusion study.

For each dilution level, other than the lowest, 36 fusions were tested. At all levels, down to 0.13% VAF, all 36 fusions were detected (Fig 3A). At the lowest level of 0.06% VAF, 90% (27/30) were detected.

To test the assay performance across a broader spectrum of fusions an additional 44 breakpoints were synthesized then spiked into fragmented genomic DNA. 18 of these were designed according to breakpoints previously reported in NSCLC cases [20]. A further 26 were generated through random joining of fusion partners (S10 Table). The 18 published breakpoints where diluted to 1% and 0.5% in fragmented DNA and analyzed across multiple operators whilst the random breakpoints were diluted to 1% VAF and analyzed a single time.

Of the 44 unique synthetic fusions analyzed, 43 were detected in all repeats at 1% VAF (the remaining fusion was detected in 1 of 4 repeats) (Fig 3B, 3C & 3D). With the inclusion of Horizon cell line DNA, 97.8% (45/46) of the gene fusions were detected in all repeats at 1% VAF. At 0.5% VAF just one additional fusion was not detected in 2 of 4 repeats. All other fusions were called in all repeats at this level (Fig 3A, 3B & 3C) resulting in a total of 90% (18/20) of gene fusions that were detected at 0.5% VAF.

To extend the assessment of fusion detection sensitivity, the 2.5% VAF Horizon cell line fusion DNA was spiked into blood from 19 different donors (collected into both Streck BCT and EDTA tubes) at levels close to our LoD as described below. All fusions were detected in these samples (S11 Table). Collectively 54 unique samples with fusions and 104 variants at or above our LoD were analyzed. All but 3 were detected giving a PPA of 97.1% (≥0.5% VAF).

### Amplification detection sensitivity, repeatability and reproducibility

To determine the sensitivity of the InVisionFirst assay to *EGFR*, *FGFR1*, *ERBB2* and *MET* amplifications, double stranded DNA matching the parts of these genes targeted by the assay was manufactured, quantified by dPCR, then spiked into a background of sheared wild type DNA creating samples with copy number amplification ratios (CNAR) of 1.25x, 1.5x and 2x. Each synthetic amplification was analyzed multiple times by three operators using two different Lots of reagents (S3 Table).

In total, each gene amplification was assessed at each dilution level between 22 and 24 times across the 3 operators. All amplifications for all four genes were detected at 2x CNAR, while 86 out of 88 amplifications were detected at 1.5x (97.7%). Detection at 1.25x CNAR ranged from 59% for *FGFR1* to 90.91% for *EGFR* (Table 1).

**Table 1:** Amplification sensitivity analysis for *FGFR1*, *EGFR*, *ERBB2* and *MET*.

Gene	1.25X (%)	1.5X (%)	2X (%)
MET	81.82	95.45	100
FGFR1	59.09	100	100
ERBB2	68.18	100	100
EGFR	90.91	95.45	100
Combined	75	97.73	100

An additional series of samples were created with amplifications of all four genes between 2x and 50x to assess our reportable range and extend our assessment of PPA. A total of 49 unique samples were analyzed with 52 variants at or above our LoD of 1.5x CNAR and 51 variants were detected giving a PPA of 98.1% (S6 Table).

### Specificity of the InVisionFirst assay

To determine assay specificity, blood was drawn into Streck BCT or EDTA tubes from donors not known to have cancer. 95 samples were analyzed for gene fusions and no calls were made. 109 samples (70 in Streck BCT and 39 in EDTA tubes) were analyzed for SNVs, indels and amplifications. No CNVs were detected in these 109 individuals. A total of 3 coding or splice altering variants were called at a VAF of between 0.13% and 1.57% (TP53 L369X, a TP53 splice alteration at chr17:7673838 and EGFR T790M, S12 Table). Digital PCR analysis was performed targeting all changes. The *TP53* mutation (g.chr17:7673838 C>A) at 1.57% was confirmed by dPCR. The mutations at 0.13% and 0.29% were not detected by dPCR. To determine the frequency with which we call these changes, we analyzed the presence of these changes in a further 92 samples from donors not known to have cancer and 242 samples from untreated NSCLC patients. None of these alterations were detected in this extended cohort.

### Comparison of the InVisionFirst assay with dPCR

**Fig 4:**
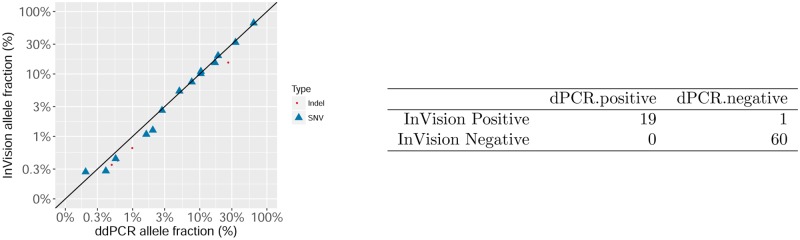
Comparison between InVisionFirst assay and orthogonal dPCR generated by an independent laboratory. Four common cancer mutations were tested by dPCR in 20 samples selected to have one of these four mutations based on the InVisionFirst assay. The allele fraction of the mutation not detected by the orthogonal method is estimated by the InVisionFirst assay at 0.3%.

To compare InVisionFirst with an orthogonal method, blood from 20 NSCLC patients was assessed with both the InVisionFirst assay and dPCR. Twenty patients were first identified with either a *KRAS* (p.G12C or p.G12D) or *EGFR* (p.L858R or p.E745_A750del/K) mutation above 0.25% VAF by the InVisionFirst assay (0.27%-65.55% VAF). In this cohort 40% of patients had a VAF <0.75% (S13 Table). cfDNA from a second tube of blood was then extracted from all 20 donors and shipped to an independent site (LGC, Teddington, UK) for blinded analysis. LGC analyzed all samples for all 4 mutations. Using dPCR they detected 19 of the 20 expected changes while not identifying any unexpected changes giving a PPA of 100% and a Positive Predictive Value (PPV) of 95% (Fig 4). The one change not detected by dPCR was a change identified at 0.3% by the InVisionFirst assay and was in a sample with comparatively low DNA input levels (an estimated 646 molecules were read by dPCR, implying 1–2 mutant molecules were to be expected in the sample). Comparison of VAF between dPCR and InVisionFirst showed excellent concordance (R-squared = 0.965) (Fig 4).

### Streck BCT and EDTA tube comparison

To demonstrate that the InVisionFirst assay can be used to analyze blood collected in either Streck BCT or EDTA tubes, reference DNA with SNVs, indels and fusions were spiked into the blood of donors not known to have cancer. 16,000 amplifiable copies of either sheared Tru-Q2, Tru-Q3 or the custom 2.5% VAF fusion cell line DNA (Horizon Discovery) were spiked into either blood tube type. Following extraction and successful sequencing of 36 spiked samples, all expected mutations were detected in both tube types (S11 Table).

### Effect of delayed processing on mutant allele fraction

**Fig 5:**
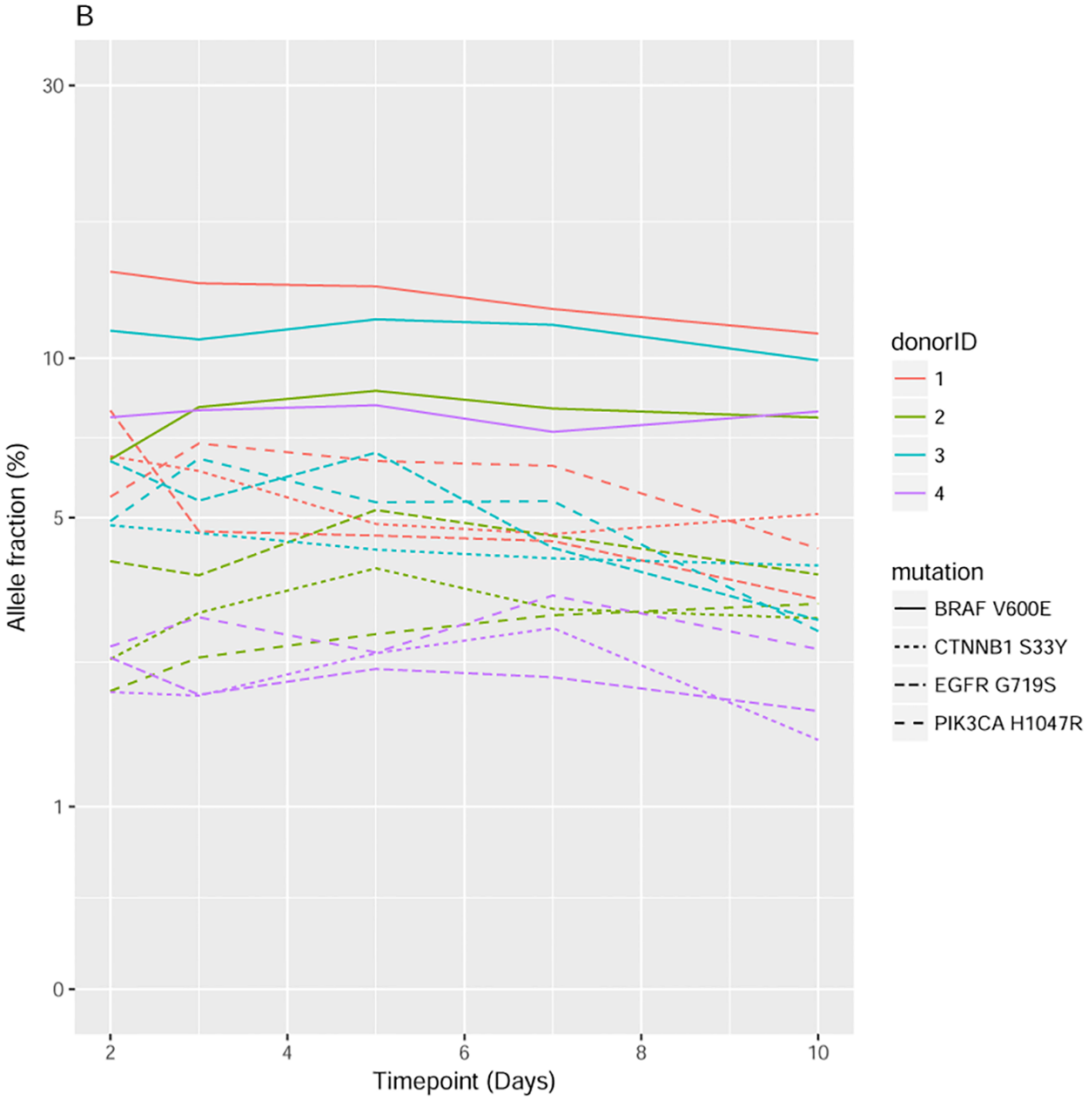
Stability of AF over time using Streck blood tubes spiked with Horizon reference material.

To assess the impact of delayed processing on mutant VAF for blood drawn into Streck BCT, Horizon 5% Multiplex I cfDNA Reference Standard DNA was spiked into whole blood from 4 donors then processed at 2, 3, 5, 7 or 10 days post blood draw. Variant allele fractions were assessed by the InVisionFirst assay and were shown to be stable following room temperature storage for up to 10 days (Fig 5).

## Discussion

A number of assays with varying performance characteristics are available for molecular stratification of patients with NSCLC. In a recent study of patients treated with osimertinib following detection of an *EGFR* T790M mutation through ctDNA sequencing, 3 of the 7 best responders had the T790M mutation detected at VAF<0.25%, highlighting the potential benefit to patients of more sensitive assays [21].

Most assays have either high sensitivity for one or a limited number of mutations, such as assays based on dPCR, or a low sensitivity for a broader spectrum of changes. Recently, a limited number of assays that aim for both broad coverage and high sensitivity were introduced such as the InVisionFirst assay. In order for clinicians to differentiate assays and determine the one most suitable for their patient, detailed analytical validation, clinical validation and clinical utility studies will be needed in combination with factors such as turnaround time, cost and reproducibility.

Here we have demonstrated that the InVisionFirst assay has exceptionally high sensitivity for detecting SNVs, indels, amplifications and gene fusions. We have also shown high SNV and indel detection concordance between the InVisionFirst assay and dPCR in blood samples from NSCLC patients. Out of 80 possible changes assessed by both methods, 79 were concordant. A single *KRAS* p.G12C change that was detected by the InVisionFirst assay at VAF of 0.3% was not observed by dPCR. Multiplexing by dPCR is typically not practical and therefore all samples in this study had to be split 4 ways to analyse the 4 mutations by dPCR. The discordant *KRAS* change was found in a sample with low concentration of DNA and due to both this splitting of material and to sample loss (dead volume) common in dPCR (~39%); we therefore only expected to see ~ 1 to 2 mutant molecules in dPCR analysis, and the probability of allelic loss (zero representative molecules in the assay) was substantial (estimated probability of at least 14.4% to have no mutant copies present according to Poisson statistics). Separately a blinded study comparing InVsionFirst with both dPCR analysis of cfDNA and sequencing of tissue has shown high concordance [22].

In the analysis of donors not known to have cancer, no fusion or CNV calls were made. Three unexpected protein coding alterations were called in analysis of 109 samples (per base specificity 99.9997%). Through dPCR analysis, one of these alterations was confirmed whilst the other two were not detected. These calls were made at low VAF (0.13% and 0.29%) and could represent either false positives or true changes that could not be replicated at such low levels. We reviewed available data from a further 344 individuals that were either not known to have cancer or were newly diagnosed, untreated NSCLC patients, and neither change was detected in these samples, so each was detected only once in >450 samples. These data confirm the specificity of the assay. While false-positive calls in cfDNA at low VAF can occur, results from clinical studies have demonstrated that patients treated with osimertinib following detection of EGFR T790M mutation in plasma down to 0.06% VAF have achieved high rates of clinical response, demonstrating the importance of detecting mutations at low VAF [21]. Additional data is required to confirm that preliminary observation in larger populations. Based on the data presented here, the clinical specificity would appear to be greater than 99%. Measures such as setting a hard threshold based on a minimal allele fraction may thus result in the loss of clinically relevant true positive calls.

In order to attain high sensitivity and specificity in NGS analysis of ctDNA a number of factors are important. Key amongst these are conversion of sufficient DNA into a sequencing library and sequencing to sufficient depth to read mutant molecules multiple times and to have a low chance of missing mutant molecules in the sequencing step. Through the use of an amplicon based strategy, we previously showed that any molecule spanned by the amplification primers should be read [12]. The use of short amplicons results in a high fraction of DNA being analyzed. Other methods for targeted NGS analysis typically incorporate ligation steps and cleanup steps prior to amplification which may result in lost mutant molecules before analysis starts.

A second challenge is sequencing depth. By focusing on a panel of 36 key genes we can sequence more deeply than is routinely achieved for many larger panels. In this study using our routine process, the median depth at which an SNV was called was ~69,000x and 95% of calls had a depth greater than half this (S5 Table). By contrast, the current target depth for Foundation ACT is >5000x unique median coverage [23] and the target depth for Guardant 360 v2.10 is 15,000x [24]. We have a reportable range down to 0.0125% VAF for indels and SNVs, and with our high sequencing depth even a mutation at this level would typically result in 9 mutant reads.

The InVisionFirst assay has been developed to analyze blood drawn into either Streck BCT or EDTA tubes. Here we have demonstrated that when mutant DNA was spiked into both tube types at close to our LoD, neither inhibited mutation detection. As we assessed our specificity using blood drawn into both tube types, we have shown there is not a significant impact on specificity. The performance of Streck BCT for preventing white blood cell lysis and subsequent reduction in mutant allele fraction has already been demonstrated by others both in pregnant donors and cancer patients [25,26]. Our results support this showing that the mutant allele fractions of spiked DNA detected by the InVisionFirst assay stayed stable when blood processing was delayed for up to 10 days.

This study demonstrates that the InVisionFirst assay has high analytical sensitivity, specificity and reproducibility which are appropriate for clinical applications. Separate studies are ongoing to test clinical validity and utility in a range of settings.

## Materials and methods

### Healthy donor and cancer patient blood collection

For analysis of assay specificity, blood was collected from donors not known to have cancer. Blood was collected by a trained phlebotomist into both Streck BCTs and EDTA tubes by BioreclamationIVT (NY, USA). A minimum of two 10mL tubes were collected from each donor. For orthogonal assessment comparing the InVisionFirst assay to dPCR, blood was collected into Streck BCT from a series of NSCLC patients. All were analyzed using the InVisionFirst assay and the first 20 of those identified as having *KRAS* (p.G12C or p.G12D) or *EGFR* (p.L858R or p.E745_A750del/K) mutations at or above our LoD (0.25% VAF) with a second tube of blood available were selected for dPCR orthogonal testing. For extended assessment of 2 locations with potential false positives (chr7:55181378 C>T and chr17:7669684 C > -), an additional group of 242 untreated NSCLC patients from the same series as above and a further 92 individuals not known to have cancer were analysed.

Institutional Review Board (IRB) approval was obtained from the six centers collecting samples (Levine Cancer Institute, University of Colorado Lung Cancer Research Center, Holy Cross Hospital, Mid-Florida Hematology and Oncology, Christiana Care and North Shore Hematology Oncology). All patients provided written informed consent and data was de-identified so no patients could be identified by study personnel outside of the clinical trial site including the study authors.

Upon collection, the Streck BCTs were gently inverted 8–10 times before being shipped immediately to Inivata Inc (North Carolina) where they were processed within 7 days of collection. Here they were centrifuged at 1600 x g for 10 minutes at room temperature, plasma was removed, transferred to a new tube and a 2^nd^ centrifugation step was performed at 20,000 x g for 10 minutes to pellet any remaining cellular debris before transferring the plasma to a new tube.

EDTA samples collected by BioreclamationIVT were processed immediately following collection before shipping to Inivata. The one significant modification from the Streck SOP was that the second spin was performed at 6500 x g as a faster centrifuge was not available. Upon completion of processing all cfDNA samples were frozen at -80 °C until ready for analysis.

### Contrived ctDNA samples

#### SNVs

To assess SNV detection performance the Horizon Tru-Q reference material was used. Tru-Q7 and Tru-Q0 DNA were both sheared to ~200bp (Covaris) and quantified by Horizon Discovery. Tru-Q7 contains 39 validated mutations targeted by the InVisonFirst assay. 32 of these are at low allele fractions with the majority between ~1%-1.3% VAF (range: <1%-30% VAF). A full list of all mutations is available in S2 Table. All genomic changes described in this and subsequent tables use the hg38 human genome build. Two-fold dilutions were performed four times using Horizon Tru-Q 0 wild-type DNA as diluent to create the following mixes: 1%-1.3%, 0.5%-0.65%, 0.25%-0.33%, 0.13%-0.16% and 0.06%-0.08% VAF.

#### Indels

The Horizon Tru-Q7 reference DNA contains just a single indel (*EGFR* del746-A750). To assess the InVisionFirst assay’s indel calling performance, SeraCare manufactured a custom indel reference material. 9 common insertions (+1 to +12bp) and 9 common deletions (-1 to -24bp) targeted by the InVisionFirst assay were synthesized by SeraCare. An additional 2 indels not currently covered by the InVisionFirst assay were also created in the mix. These mutations were mixed against “Genome in a Bottle” (GM24385) wild-type genomic DNA to produce mixes where the 18 targeted indels were present at approximately 2%, 1%, 0.5%, 0.25% or 0.1% VAF. The DNA was then sheared to ~150bp (Covaris) and the top three dilution levels were assessed by dPCR by SeraCare in order to confirm each indels VAF as compared to wild type background DNA (S7 Table). The lowest two dilutions were not tested by dPCR due to the expected low VAF of the indels.

#### Fusions

The InVisionFirst assay identifies *ALK* and *ROS1* gene fusions by detecting the genomic breakpoint junctions that bring the relevant genes together. As relatively few fusion-associated DNA breakpoints have been published to date and as there are only a small number of reference materials and cell lines with published *ALK* and *ROS1* DNA breakpoints, three different contrived materials were used.

A custom cell line mix was generated by Horizon Discovery. The resultant mix contained 1 *ROS1* and 1 *ALK* fusion (S10 Table). Following shearing by Horizon Discovery, they demonstrated with dPCR that the two fusions were present at ~2.5% VAF. These were subsequently diluted to 1%, 0.5%, 0.25%, 0.12% and 0.06% VAF.

To further assess the performance of the assay over a broad spectrum of breakpoints, the DNA junctions from 8 published *ROS1* gene fusions and 10 published *EML4*-*ALK* gene fusions were identified [20]. A further 26 synthetic fusions were then designed by computationally joining a 5’ and 3’ partner anywhere randomly within the common introns and exons targeted by the InVisionFirst assay (S10 Table).

A 500 bp sequence was designed for all fusions with the DNA breakpoint in the center. Synthetic fusions were manufactured as double stranded DNA fragments (IDT) then diluted and sheared (Covaris) to ~160 bp before dPCR quantification. Genomic DNA (Bioline) was also sheared to 160 bp and quantified using dPCR targeting a 108 bp region of the *RPP30* gene. The patient specific fusion DNA fragments were then spiked into genomic DNA at two different levels (1% VAF and at 0.5% VAF). Eight samples were created with both an *ALK* and a *ROS1* fusion and a further two were created with just a single *ALK* fusion. Thirteen samples were created containing one of the randomly generated *ALK* fusion sequences and one of the *ROS1* fusion sequences. All were present at 1% VAF (S10 Table).

#### CNVs

In order to assess the InVisionFirst assay’s performance for CNV detection, a similar approach was taken to our analysis of fusions. Firstly, the amplicons we use for targeting the 4 genes currently assessed for amplification by the InVisionFirst assay were identified (*EGFR*, *FGFR1*, *ERBB2* and *MET*). 160 bp fragments of DNA were then selected encompassing these regions. These were synthesized as double stranded DNA fragments (IDT) then quantified by dPCR. Genomic DNA (Bioline) was then sheared and quantified as above to use for dilutions.

Each quantified double stranded DNA fragment was pooled by gene such that each targeted region was equally represented (for example *EGFR* had 11 targeted regions synthesized). Sheared background DNA and CNV pools were then combined to give the relevant amplified amounts.

#### Extended assessment of PPA and PPV

To further assess the InVisionFirst assay’s ability to call a broad spectrum of SNVs, indels, fusions and CNVs across a large range of allele fractions above our LoD, an additional series of undiluted and diluted samples were created using DNA from the Horizon Tru-Q reference material series and SeraCare reference DNA (SeraCareTriLevel and SeraSeq). An extended set of CNV samples were created with CNAR of between 2-50x using the same method outlined above and mutant DNA was also spiked into a range of healthy donor samples as described below. Each of these samples was run just once. A full table of the different samples and their detected mutations is available in S6 Table.

### Library preparation and analysis with the InVisionFirst assay

An earlier version of this assay based on eTAm-Seq has previously been described [17]. The InVisionFirst assay is based on the same approach but with the addition of the ability to call gene fusions. The updated assay also has an updated primer panel adding coverage to key *ALK* and *ROS1* inhibitor resistance mutations, and an amplicon size distribution of 73bp-155bp (median = 112bp). The targeted exons and introns targeted for fusion detection are described in S1 Table. cfDNA was first extracted from plasma using the QIAamp Circulating Nucleic Acid kit (Qiagen) followed by quantification by dPCR using the BioRad QX200 and an assay targeting a 108 bp region of the ribonuclease P/MRP subunit p30 (*RPP30*) gene. Contrived samples were quantified using the same assay. Yields were expressed as amplifiable copies (AC) of DNA. Two separate libraries were then setup in parallel from two blood tubes or from the contrived DNA. Where libraries were prepared using contrived samples, 16,000 amplifiable copies of the genome were used except in the amplification study where a mix of 16,000 and 2000 amplifiable copies (minimum input) were used. Both libraries were setup using a two-step amplification process that first targeted the desired regions then incorporated replicate and patient-specific barcodes and Illumina sequencing adaptors (See Fig 1). The first library targets SNVs, indels and CNVs whilst the second library has been designed to target all introns and exons brought together to create the three major *EML4*-*ALK* variants which collectively account for 93% of ALK fusions found in the COSMIC database (COSMIC Version 83). It also targets 92% (COSMIC V83) of the intronic and exonic bases brought together to create *CD74*-*ROS1*, *SLC34A2*-*ROS1*, *SDC4-ROS1* and *EZR-ROS1* fusions in lung carcinomas (S1 Table).

For both library types up to 48 samples were pooled together including positive and negative controls before sequencing on the Illumina NextSeq 500 (300 cycle PE) with 5% PhiX to monitor sequencing performance. Sequencing files were analyzed using the Inivata Somatic Mutation Analysis (ISoMA) pipeline to identify SNVs, CNVs and indels and the FUSP pipeline to call fusions. For the ISoMA pipeline a minimum Phred quality score of 30 for each base was required for inclusion in the analytics. In each run, in addition to the controls, we used the non reference allele fraction at common single nucleotide polymorphisms (SNPs) to detect potential contamination events. In addition, the overall sequencing depth at these common polymorphisms was used as part of quality control to confirm that sufficient sequencing depth had been generated.

For SNV and indel analysis, a background model was first established using samples from presumed healthy donors for each position/base pair change covered by our panel. The final determination of an SNV call integrated the data across multiple replicates for each sample in comparison with this background within a maximum likelihood framework. The same statistical principle was used for indels using samples from the same analytical batch in order to enable appropriate background calibration. The minimum depth at which any SNV or indel would be called was 1000x. In order to identify CNVs, a normalized measure of read depth was generated correcting for sample and amplicon effects in order to infer the copy number ratio between the 4 assessed genes (*ERBB2*, *FGFR1*, *MET* and *EGFR*) and the remainder of the genome.

Fusions were called by identifying the breakpoint sequences created when fusion partners joined. Patients with sequence reads matching to a 3’ and 5’ fusion partner were identified as fusion positive (e.g. *EML4* intron 13 and *ALK* intron 19). When the same breakpoint is detected twice, a fusion call is made. All variants were annotated using the canonical transcript for each gene. All SNVs and indels that resulted in coding and splice-site mutations were reported. Finally, a mutation calling report was generated providing a comprehensive summary of somatic alterations identified.

### Orthogonal dPCR analysis

20 patients with NSCLC in whom a *KRAS* (p.G12C or p.G12D) or *EGFR* (p.L858R or p.E745_A750del/K) mutation was detected by the InVisionFirst assay above our LoD (0.25% VAF), were selected for dPCR orthogonal testing as described above. DNA was extracted from a second tube of blood from all 20 patients, assessed by dPCR using the *RPP30* assay then shipped on dry ice, anonymized, to LGC.

LGC had previously determined the suitability of the dPCR assays targeting the four mutations on the BioRad QX200 using both commercially available cfDNA standards (Horizon Diagnostics) and a set of in-house materials (*KRAS* G12C). The LoD for each assay was calculated at the start of the study using the false positive rate determined from ≥4 dPCR reaction per assay using ~116ng wild type gDNA per reaction. (S14 Table). Importantly although this LOD is achievable in samples with 116ng of DNA or greater, in samples with lower DNA inputs, sensitivity will be reduced in a predictable fashion based on the stochasticity of small numbers of mutant molecules.

LGC then performed a single dPCR with the *KRAS* G12C assay to determine the DNA concentration and whether the DNA could be run undiluted or needed to be diluted to be within the dynamic range of the BioRad QX200 dPCR platform. Finally, DNA from all twenty patients was assessed in triplicate (7μl per reaction) using the four assays and samples were called mutant or wild type depending on whether they were above or below the assays LoD (S13 and S14 Tables).

### dPCR assays designed for assessment of unexpected calls (potential false positives)

In order to validate possible false positive calls made during the analysis of healthy donors, two digital PCR assays were ordered from BioRad and one was kindly donated by Dr Dana Tsui (Cancer Research UK Cambridge Institute, University of Cambridge, UK). The assays that were sourced from BioRad were designed using their online tool (https://www.bio-rad.com/digital-assays/#/). Synthetic mutant sequence for each assay was also designed using the online tool, and were ordered as double stranded DNA (IDT) and delivered pre-diluted (to 2000 copies per ul in 10mM Tris pH 8, 0.1mM EDTA and 0.1mg/mL Poly A). A mix of wild type DNA (BioLine) and ~5% synthetic mutant DNA was first tested with all 3 assays using a temperature gradient to determine optimal annealing temperatures. A dilution series of mutant to wild type DNA was then created then run in duplicate at two different concentrations along with wild type DNA to determine the background and limit of detection of each dPCR assay. Finally, each sample was run at least 4 times using 5μl of DNA.

### Robustness of the InVisionFirst assay to Streck BCT and EDTA blood tube collection

To test that the InVisionFirst assay gives comparable results whether blood is collected in Streck BCT or EDTA blood tubes, blood from multiple donors was drawn into each tube type then this was processed to plasma as described above.

Both Streck and EDTA plasma was spiked with 16,000 amplifiable copies of either the Horizon fusion cell line reference material at 2.5% VAF or sheared Horizon reference material Tru-Q2 or Tru-Q3 which contain up to 14 variants (SNVs and indels) at 4 to 30% VAF. All samples were then mixed, extracted, then analyzed using the InVisionFirst assay.

### Effect on mutant allele fraction of delayed Streck BCT processing

Whole blood was collected into five 10mL Streck BCTs from four individual donors as described above by BioreclamationIVT. This was shipped to Inivata and upon receipt of the tubes, 4000 copies of sheared (200bp) Horizon 5% Multiplex I cfDNA Reference Standard (Horizon Discovery) DNA was spiked into each sample. The five tubes from each donor were then randomized and kept for 2, 3, 5, 7 or 10 days at room temperature (~26°C) before routine processing and analysis by the InVisionFirst assay. The allele fraction of each of the detected mutations from the Horizon reference standard was then compared to the matched sample from day 2 as a baseline to determine reduction in mutant VAF induced by delayed processing.

## Supporting information

S1 Table Details of the 36 genes targeted by InVisionFirst and which variant types are assessed for each gene. All exons covered for SNV, indel and CNV analysis are described. All introns and exons covered for fusion gene detection are described.
(XLSX)

S2 Table Tru-Q7 (Horizon) reference DNA mutations. A full list of validated mutations covered by the InVisionFirst panel are listed along with Horizon’s validated variant allele fraction. All 39 variants are grouped by type.
(XLSX)

S3 Table Overview of each study used to assess the four different variant types limit of detection. (XLSX)

S4 Table SNV sensitivity at different dilution levels of Horizon Tru-Q7 DNA. (XLSX)

S5 Table Full list of all SNVs in the Tru-Q7 dilution study and whether they were detected. DNA input (as assessed by dPCR), detected variant allele fraction (VAF) and total sequencing depth are all described.
(XLSX)

S6 Table Details of samples used in CNV sensitivity analysis and the extended analysis of PPA for the four variant types. Section A describes the calculations used to determine PPA for all 4 variant types. Section B details the cell line mixes created and calls made in the extended assessment of PPA. Section C describes all contrived CNV samples and whether a call was made. This includes both the samples used to assess the assays LoD and those used for the extended assessment of PPA.
(XLSX)

S7 Table List of all targeted indels in the custom SeraCare indel control material and comparison of SeraCare dPCR VAFs with average VAFs determined by InVisionFirst. (XLSX)

S8 Table Indel sensitivity at different dilution levels in the custom SeraCare indel control material. (XLSX)

S9 Table Full list of all indels in the custom SeraCare indel control material dilution study and whether they were detected. DNA input (as assessed by dPCR) and detected variant allele fraction (VAF) are both described.
(XLSX)

S10 Table Details of all fusion reference materials. Part A describes the Horizon cell line mix. Section B describes the full sequence of the published patient specific fusions and section C describes all the randomly generated fusions.
(XLSX)

S11 Table List of samples spiked with mutation positive DNA (Tru-Q2, Tru-Q3 or the custom 2.5% VAF fusion cell line DNA) and whether each mutation was detected. For SNVs and the one indel a detected VAF is reported.
(XLSX)

S12 Table List of variants detected in donors not know to have cancer. (XLSX)

S13 Table List of variants detected by both InVisionFirst and blinded dPCR in an orthogonal analysis study. (XLSX)

S14 Table LoD for the 4 digital PCR assays analyzed by LGC in samples with optimal DNA input amounts. (XLSX)
